# Assessment of the VDW interaction converting DMAPS from the thermal-motion form to the hydrogen-bonded form

**DOI:** 10.1038/s41598-019-49352-1

**Published:** 2019-09-11

**Authors:** Masae Takahashi, Hiroshi Matsui, Yuka Ikemoto, Makoto Suzuki, Nobuyuki Morimoto

**Affiliations:** 1 Graduate School of Agricultural Science, Tohoku University, Sendai, 980-8572 Japan; 2 Graduate School of Science, Tohoku University, Sendai, 980-8578 Japan; 3 Japan Synchrotron Radiat. Res. Inst. JASRI SPring-8, Sayo, Hyogo 679-5198 Japan; 4 Graduate School of Engineering, Tohoku University, Sendai, 980-8579 Japan

**Keywords:** Chemical physics, Computational chemistry

## Abstract

Assessment of van der Waals (VDW) interactions is fundamental to all of the central quest of structure that regulates the biological function. VDW interactions contributing to intramolecular weak hydrogen bonding are regarded as an important force to regulate the thermal stimuli-sensitive function of sulfobetaine methacrylate, DMAPS. We present here the conversion from the thermal-motion form at room temperature to the weak-hydrogen-bonded form against thermal motion as a terahertz spectral change with a definite isosbestic point from an absorption peak of one form to the other. Vibrational absorptions are used as a probe for assessing VDW interactions in conjunction with highly reliable and well-established density functional theory (DFT) calculations for analysis. Complicated spectral features and uncertain conformations of DMAPS in the amorphous state are clearly resolved under the polarizable continuum model and the dispersion correction for the pure DFT calculations.

## Introduction

The van der Waals (VDW) interaction has become a hot topic in recent years as a constructing force of promising materials such as the VDW heterostructure^1–4^, quantum liquid^5,6^, the VDW bonded magnet^7^, molecular diode^8^, and Rydberg gas^9^. The VDW interaction named in honour of van der Waals, who first introduced attractive interactions between neutral molecules in his equation of state, is a dispersion interaction of pure quantum physical origin^10–14^. The VDW force is well known as attractive at smaller molecular separations, while the sign of the VDW force can be changed from attractive to repulsive by suitable choice of interacting materials immersed in a fluid at larger distances^15^. The VDW forces are very weak and easily disturbed by the thermal energy around room temperature. In biological systems that function at room temperature, VDW interactions provide fantastic characteristics such as fuzzy control in the function of biomolecules. It is significant to directly assess the VDW interactions. How do we determine the efficiency of a VDW interaction? The influence of curvature strain and VDW force on the interlayer vibrational mode of WS_2_ nanotubes was reported as a redshift by 2.5 cm^−1^ with confocal micro-Raman spectroscopy^16^. The vibration mode is a promising probe for assessing VDW interactions and can be used in various materials, including biological systems. In particular, terahertz (THz) vibration is powerful for detecting weak interactions^17–19^. Combined with highly reliable and well-established density functional theory (DFT) calculations for the analysis of vibrational absorption spectra, the effect of dispersive interactions can be directly revealed by the observation of vibrational spectral change at low temperature where thermal disturbance is suppressed and the VDW interaction is effective against thermal motion.

The polymerized sulfobetaine methacrylate exhibits a thermal stimuli-responsive function where intramolecular VDW interactions have a decisive role. A sulfobetaine methacrylate, DMAPS (3-[dimethyl-(2-methacryloyloxyethyl) ammonium] propane sulfonate) studied here, is an electrically neutral zwitterionic betaine monomer used in the synthesis of biocompatible polysulfobetaines. The development of biocompatible nanoparticles using zwitterionic polymers is increasingly demanded^20–24^, and we have investigated several biocompatible nanoparticulate drug delivery systems^25,26^. The biocompatibility and the thermal stimuli-responsive function of polysulfobetaines is stemmed from the zwitterionic sulfobetaine monomer. Betaine is a specific type of electrically neutral zwitterion that contains a pair of cationic functional group bearing no hydrogen and anionic functional group at non-adjacent position in the molecule. It is noteworthy that betaine is in an important position to explore the correlation between the structure and function in biological systems, because the special zwitterionic structure of betaine is biomimic of fundamental biomolecules such as amino acids, proteins, and phospholipids.

THz peaks from the methacryloyl moiety of DMAPS are predicted to be observed even in the amorphous state^27^. The molecular size of DMAPS is small enough to calculate with reasonable accuracy and sufficient level. DMAPS contains intramolecular weak hydrogen bonds predominantly governed by dispersion interactions such as the VDW force, and the formation of the intramolecular weak hydrogen bonds at low temperature is expected to affect the THz vibrations. Amorphous samples are more informative from the biological point of view and the THz spectroscopic study of amorphous samples is desired. However, the study of amorphous samples is lagging behind. A small molecule in an amorphous state gives no spectral feature in the THz spectral region, although a molecule in a crystalline state gives THz peaks^28^. Early THz spectroscopic studies of proteins provided THz peaks even in noncrystalline states^29^, but the peak assignment of proteins is difficult due to the large molecular size. Another issue in the THz spectroscopic study of amorphous system is the treatment of surrounding molecules in the calculation for peak assignments. We have studied intermolecular weak hydrogen bonding of crystalline compounds using THz spectroscopy with DFT calculations^30–33^ and synchrotron FTIR microspectroscopy^34^. Nowadays, THz spectroscopic studies are well established for crystalline systems including microcrystalline powder samples and THz peaks due to intermolecular weak hydrogen bonding networks are obtained as coupled with intermolecular translation and rotation modes. THz peaks are very sensitive to the environment of respective molecule, and even the assignment of intramolecular modes in crystalline compounds requires periodic solid-state calculations that include the effects of surrounding molecules in crystals.

**Figure 1:**
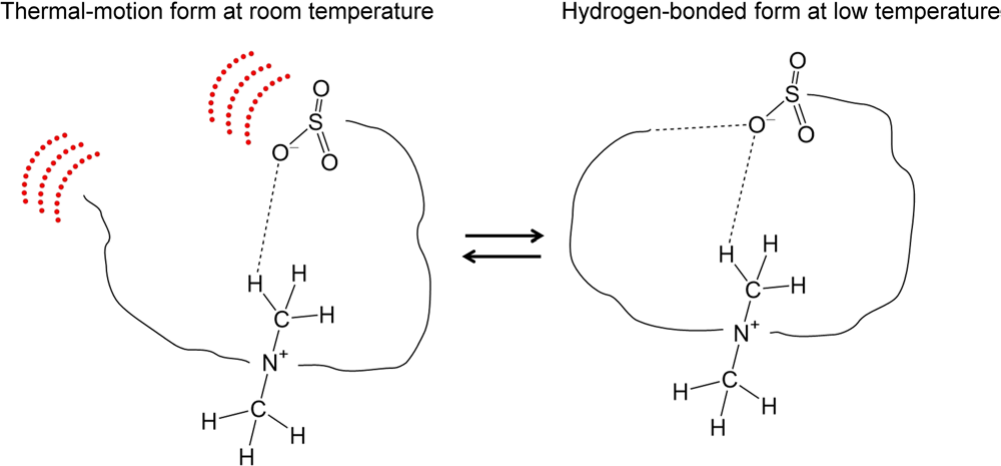
Thermal-motion form of DMAPS at room temperature and hydrogen-bonded form at low temperature.

To assess the VDW interaction, we study herein the low temperature behaviour of the biocompatible DMAPS molecule in the vibration absorption of THz spectroscopy and synchrotron FTIR microspectroscopy. Weak hydrogen bonds found in DMAPS are one of three classes of hydrogen bonds ranging from weak (VDW limit) to strong (covalent-bond limit) depending on strength^35^, and the dispersion forces that contribute mainly to the weak hydrogen bond are one of three interactions (electrostatic, induction, and dispersion) contributing to hydrogen bonding. Dispersive interactions, such as VDW interactions and weak hydrogen bonds, are easily disturbed by thermal energy near room temperature (thermal-motion form in Fig. 1), whereas thermal disturbance is suppressed at low temperature, where weak hydrogen bonds are formed against thermal motion (hydrogen-bonded form in Fig. 1). Here we focus on the very weak hydrogen bond formation (hydrogen-bonded form) and cleavage (thermal-motion form) at the VDW limit, where the thermal-motion forms dominate at room temperature. Some hydrogen bonds remain in both hydrogen-bonded and thermal-motion forms. The spectral change in vibrational absorption is predicted to be a change between the thermal-motion and hydrogen-bonded forms. We perform a gas-phase calculation for peak assignment instead of a solid-state calculation because the sample studied here is fully amorphous, Here, we incorporate the dielectric constant into the gas-phase calculation as the effect of the surrounding molecules with a large dipole moment, and perform the dispersion correction for the pure DFT calculation to describe the weak hydrogen bond.

## Results

### Low-temperature behaviour of DMAPS in the far-infrared spectrum

**Figure 2:**
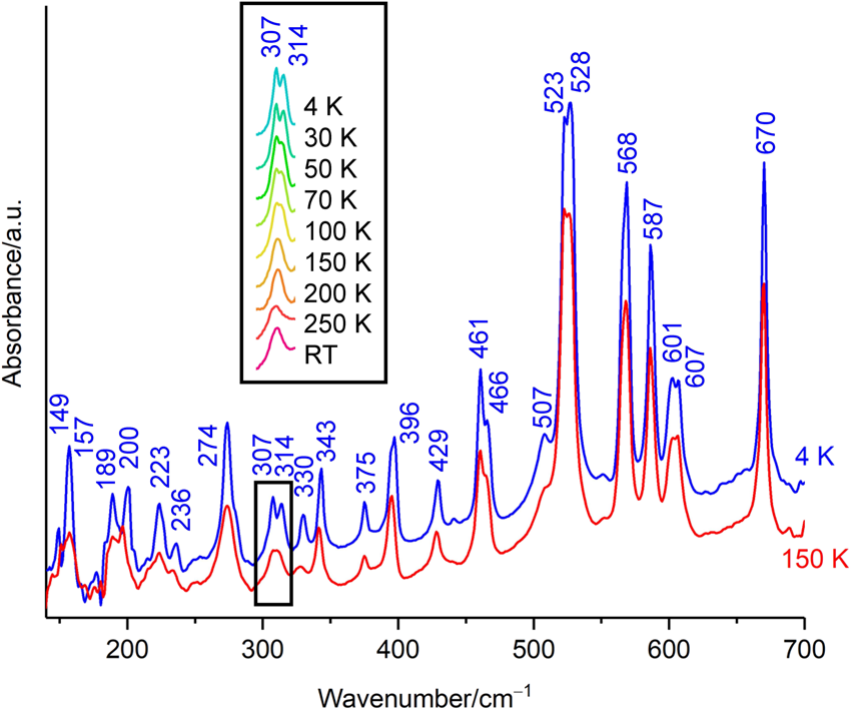
Far-infrared spectrum of DMAPS at 4 K in blue and 150 K in red. The temperature dependence at approximately 300 cm^−1^ from room temperature (RT) to 4 K indicated by the square box is given in detail in the insert. The frequency of each peak measured at 4 K is given in cm^−1^.

The far-infrared (FIR) spectral profile of DMAPS in the range from 140 to 700 cm^−1^ was measured by synchrotron FTIR microspectroscopy to investigate the behaviour at low temperature where VDW interactions become effective. The overall spectral profile hardly changes upon cooling from room temperature to 4 K, except for peak sharpening and a slight peak shift to high frequency. The spectra from 140 to 700 cm^−1^ at 150 K and 4 K are shown in Fig. 2. Remarkable difference between the two spectra was observed at approximately 300 cm^−1^. One peak at 150 K splits in two at 307 and 314 cm^−1^ at 4 K (see the square box in Fig. 2), presumably indicating the structural change between the thermal-motion form and hydrogen-bonded form shown in Fig. 1. The insert of Fig. 2 shows the details of the temperature dependence around 300 cm^−1^ from room temperature to 4 K. It can be seen from the insert of Fig. 2 that the peak splitting occurs clearly below 100 K. It is found from the temperature dependence of the full width at half maximum (FWHM) that the FWHM suddenly decreases at 100 K (14.5 cm^−1^) to 150 K (12.6 cm^−1^) (Supplementary Fig. S1). Thus, temperature-dependent spectral changes are judged not as thermal broadening but as peak splitting. The thermal-motion form is dominant at 150 K or more as characterized by one peak and the hydrogen-bonded form dominant at 100 K or less as characterized by two peaks. A reversible temperature-dependent spectral change was obtained between the cooling process from room temperature to 4 K and the heating process from 4 K to room temperature. It is well known that conformational differences are distinguished from each other in the FIR frequency region. The various conformations possible for DMAPS showed considerably different spectral patterns in our DFT calculations. Therefore, no remarkable spectral change from room temperature to 4 K suggests no conformational change due to cooling. In addition, the possibility of a conformational mixture or a dimer formation is excluded due to the number and sharpness of the measured peaks, although the conformation in the amorphous powder of DMAPS is unknown.

**Figure 3:**
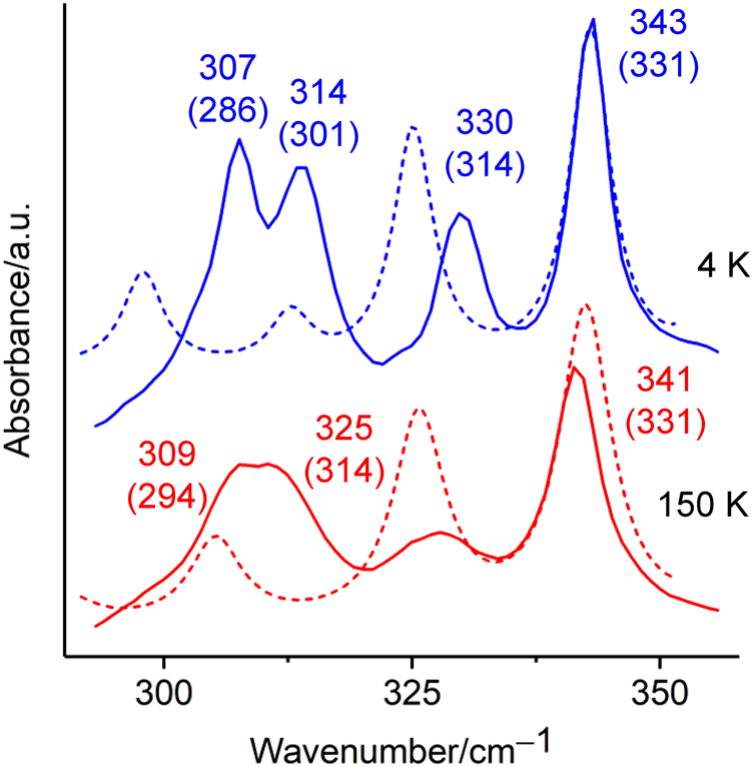
Experimental far-infrared spectra of DMAPS at 150 K and 4 K around 300 cm^−1^ (solid lines) and calculated spectra using dispersion-corrected DFT under the PCM model with dielectric constants (*ε*) of 15.20 (red dashed line) and 8.33 (blue dashed line). The FWHM of the calculated spectrum is set to the same value as that of the strongest peak in this frequency region (6.4 and 5.0 cm^−1^, respectively). The calculated spectrum is shifted by 12 cm^−1^ so that the strongest peaks of the blue dashed and solid lines coincide. The experimental and calculated frequencies (in parentheses) of respective peak are given in cm^−1^.

**Figure 4:**
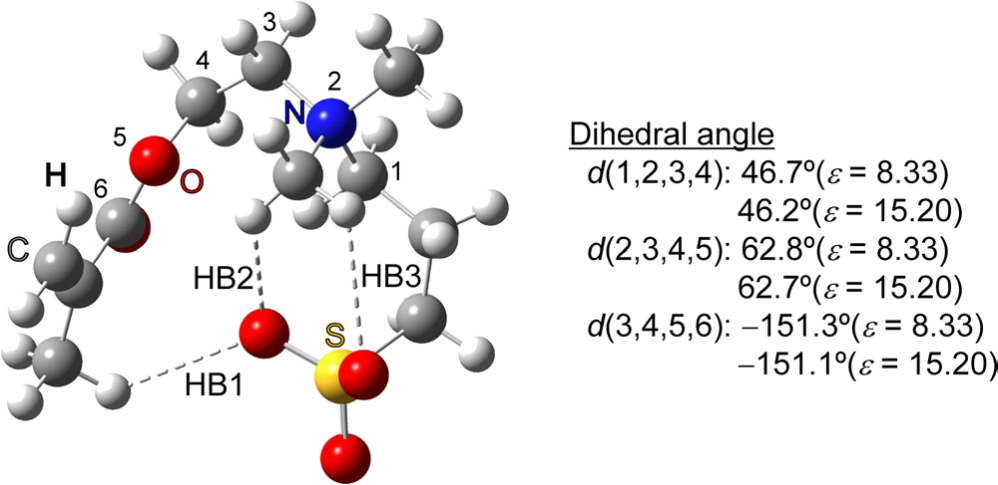
DMAPS conformer giving the best-fit FIR spectrum under the PCM model at the B3LYP/aug-cc-pVTZ level with dispersion correction. The atoms characterising the selected conformation are numbered from 1 to 6, and their dihedral angles at *ε* = 8.33 and 15.20 are shown. HB1, HB2, and HB3 are the O…H hydrogen bonds showing remarkable differences by changing the dielectric constant.

Figure 3 shows the calculated FIR spectra together with the experimental ones around 300 cm^−1^ where the temperature-dependent peak splitting was experimentally observed in Fig. 2. The calculated FIR spectra in Fig. 3 are those of the conformer giving the best-fit FIR spectrum to the experimental 4 K result both in frequency and intensity, among the spectra of stable conformations in Gibbs free energy at 298 K within the 2 kcal/mol accuracy of highly accurate CBS-Q (see Supplementary Table S1 for thermodynamic data). The second most stable conformer was selected as that giving the best-fit FIR spectrum (the optimized dihedral angles representing the selected conformation are given in Fig. 4). The spectrum calculated under the polarizable continuum (PCM) model^36^ with a dielectric constant of 8.33 excellently reproduces four peaks around 300 cm^−1^ (see the blue solid and dashed lines in Fig. 3): 307, 314, 330, and 343 cm^−1^ (observation at 4 K); 286, 301, 314, and 331 cm^−1^ (calculation with a dielectric constant of 8.33). The discrepancy between the calculated frequency and the experimental value of Fig. 2 is small enough (less than 10 cm^−1^) in the range of 140–250 cm^−1^, while the deviation of the calculated value from experimental result in the range of 250–700 cm^−1^ is relatively large and always negative (up to 32 cm^−1^) (see Supplementary Table S2). This is probably a hypsochromic shift due to packing and/or repulsive interactions with surrounding molecules. The temperature-dependent spectral change around 300 cm^−1^ from four peaks at 4 K to three peaks at 150 K is reproduced by changing the dielectric constant from 8.33 to 15.20 in the PCM model (dashed lines in Fig. 3). The difference of the dielectric constant affects scarcely the spectral profile in the whole range of 140–700 cm^−1^ except for the two peaks. It is reasonable that the spectrum of higher dielectric constant (15.20) exhibits the higher temperature spectrum, because the increase of temperature raises the dielectric constant due to the higher mobility of molecules caused by the cleavage of hydrogen bond at higher temperatures.

The number of peaks near 300 cm^−1^ (N) changes from 5 to 3 as the dielectric constant increases (Table 1). N = 4 and 3 correspond to the spectra below 100 K (low-temperature model) and above 150 K (room-temperature model), respectively. The spectral features (frequency and intensity) around 300 cm^−1^ are almost the same when N is the same. On the other hand, the frequencies of *ν*_1_, *ν*_2_, and *ν*_3_ in the THz spectral region hardly change when N = 4, but decrease with the increase of the dielectric constant when N = 3. Since the frequencies of *ν*_1_ are closest to each other and reproduce the stationary features of *ν*_1_ in the THz spectrum, ethyl methanoate (*ε* = 8.33) and 2-pentanone (*ε* = 15.20) are selected as solvents for the low-temperature model below 100 K and high-temperature model above 150 K, respectively.

**Table 1:** Effects of dielectric constants (*ε*) on the number of peaks around 300 cm^−1^ (N), bond distances of intramolecular hydrogen bonds (HB1, HB2, and HB3), and vibrational absorption frequencies of the first three vibrations (*ν*_1_, *ν*_2_, *ν*_3_) calculated at the B3LYP/aug-cc-pVTZ with dispersion correction.

N	*ε*	Solvent	HB1/Å	HB2/Å	HB3/Å	*ν*_1_/cm^−1^	*ν*_2_/cm^−1^	*ν*_3_/cm^−1^
3	46.83	Dimethyl sulfoxide	2.93	2.57	3.05	16(3)	27(8)	36(18)
	20.49	Acetone	2.83	2.53	2.93	23(5)	26(6)	41(13)
	15.20	2-Pentanone	2.79	2.51	2.89	31(8)	35(6)	43(16)
4	10.13	Dichloroethane	2.77	2.48	2.82	35(11)	43(8)	48(9)
	8.93	Dichloromethane	2.77	2.47	2.80	34(11)	44(7)	49(8)
	8.33	Ethyl methanoate	2.77	2.46	2.79	33(11)	43(6)	49(9)
5	2.91	Dipropylamine	2.56	2.45	2.42	33(7)	55(9)	56(3)
	2.23	Carbon tetrachloride	2.54	2.41	2.39	18(7)	49(1)	57(10)
	1.43	Argon	2.49	2.38	2.27	25(5)	47(5)	55(5)

For three intramolecular hydrogen bonds, HB1, HB2, and HB3, see Fig. 4. The IR intensities are given in km/mol in parentheses. Solvent keywords in SCRF = Solvent option are indicated as solvent.

### Structural difference with the difference of dielectric constant

Full geometry optimization was performed for the conformer giving the best-fit FIR spectrum to the experimental 4 K result, using a different dielectric constant, *ε,* under the PCM model at the B3LYP/aug-cc-pVTZ level with dispersion correction. Among 27 conformations for atoms 1–6 in Fig. 4, we obtained 24 conformers. The optimized geometric parameters hardly change between different dielectric constants except for three hydrogen bond distances, HB1, HB2, and HB3 (see Fig. 4). The conformations of the two optimized structures used as low-temperature model (*ε* = 8.33) and high-temperature model (*ε* = 15.20) are the same (see the dihedral angles of two models in Fig. 4). The different bonding nature of HB1 depending on the N value indicates that HB1 plays a decisive role in controlling the number of peaks around 300 cm^−1^ (Table 1). At the lowest three *ε*′*s* where N = 5, the distance is the typical O…H distance between the CH donor and the >S = O accepter (2.49~2.56 Å)^37^. The distance gradually increases and its bond strength weakens as the dielectric constant increases and the O atom accepter basicity decreases. At the highest three *ε*′*s* where N = 3, the distance is much more than VDW contact (>2.79 Å)^38^ and no hydrogen bonding exists. At the middle three *ε*′*s* where N = 4, HB1 gives a constant value (2.77 Å) suggesting VDW contacts probably due to quantum fluctuations. In that sense, HB1 at the middle three *ε*′*s* is a weak hydrogen bond at the VDW limit. For all dielectric constants, HB2 gives a typical O…H distance between the CH donor and the > S = O accepter (2.38~2.57 Å)^37^, and the permittivity dependence is the same as HB1 at the lowest three *ε*′*s*. Even at the middle three *ε*′*s* where N = 4, the HB3 distance is greater than 2.79 Å and no hydrogen bonds already exist in this dielectric constant range.

### THz spectra of DMAPS

**Figure 5:**
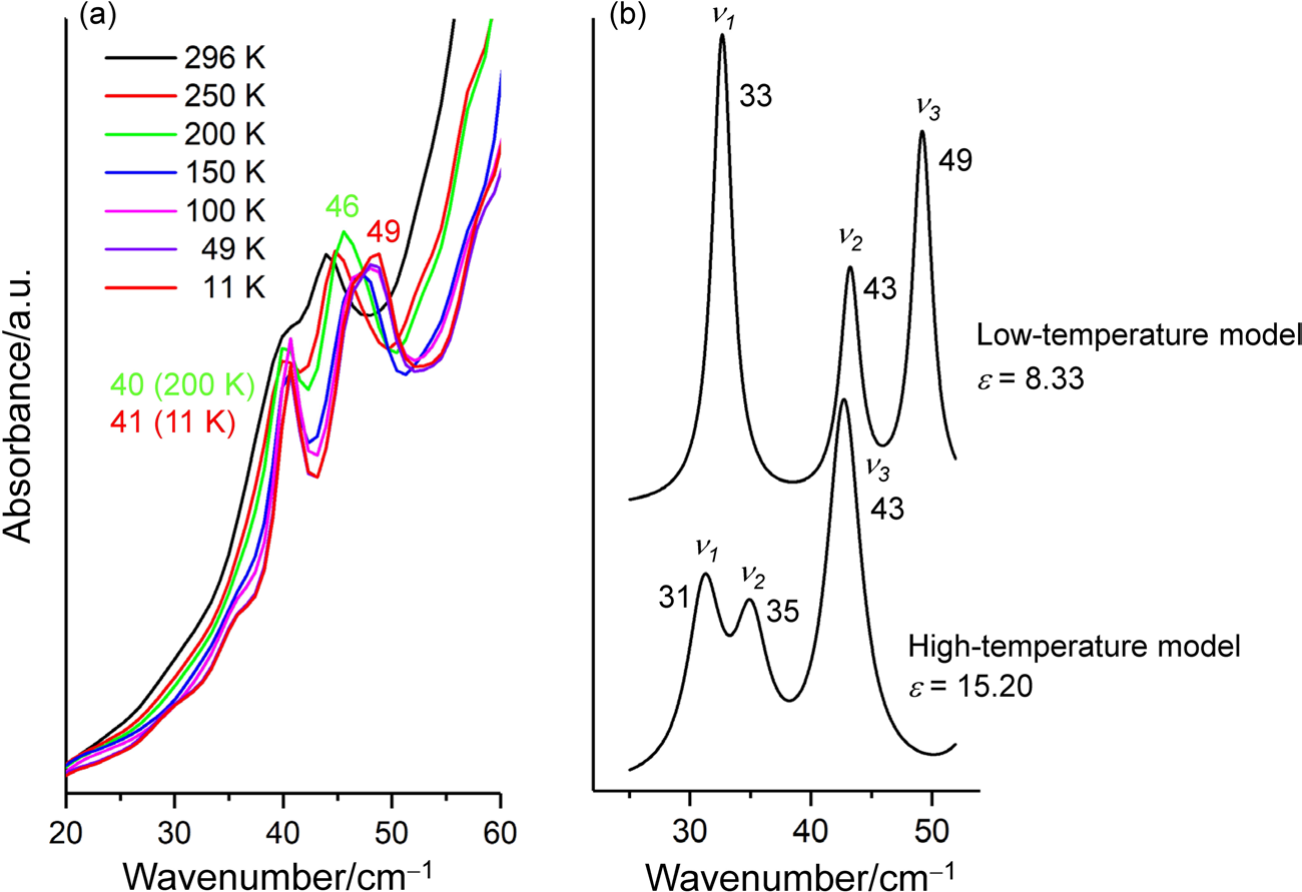
(**a**) Temperature-dependent THz spectra of DMAPS and (**b**) the calculated THz spectra of the first three vibrations (*ν*_1_, *ν*_2_, *ν*_3_) using the different dielectric constants (*ε*) under the PCM model at the dispersion-corrected B3LYP/aug-cc-pVTZ level. The FWHM of the calculated spectrum is set to the same value as that of the lowest peak (2.0 cm^−1^ for *ε* = 8.33 and 3.4 cm^−1^ for *ε* = 15.20, respectively). The calculated and the observed frequencies are given in cm^−1^.

The vibrational absorption spectrum in the THz region (20–60 cm^−1^) shows a clearer temperature-dependent spectral change (Fig. 5a), presumably indicating the structural change between the thermal-motion form and hydrogen-bonded form shown in Fig. 1. A reversible temperature-dependent spectral change was obtained between the cooling process from room temperature to 11 K and the heating process from 11 K to room temperature. At room temperature (296 K), a broad peak with a shoulder was observed (black line in Fig. 5a). By cooling to 200 K, the two peaks sharpen (40 cm^−1^ and 46 cm^−1^), and the stronger peak shifts remarkably to higher frequency (green line in Fig. 5a). Peak sharpening and high frequency shifts due to the anharmonicity of the potential energy surface in the cooling process are common in the THz spectral region^27,33,39–41^. The peak position at 40 cm^−1^ changes little by further cooling, while the peak at 46 cm^−1^ moves to 49 cm^−1^ with an isosbestic point in-between. The intensity of the peak at 49 cm^−1^ decreases compared to that of the peak at 46 cm^−1^, leaving a shoulder remaining on the low frequency side of the peak at 49 cm^−1^.

The spectral change is interpreted as the difference of dielectric constant under the PCM model (Fig. 5b). The calculation with the dielectric constant of 15.20 (high-temperature model) gives three peaks at 31, 35, and 43 cm^−1^. The frequency and relative intensity excellently agree with the observed spectra at 200 K. That is, the frequency difference is less than 10 cm^−1^ and *ν*_3_ is the strongest peak. Two peaks *ν*_1_ and *ν*_2_ may have produced one broad peak centred around 33 cm^−1^. By lowering the dielectric constant to 8.33, *ν*_2_ and *ν*_3_ shift to higher frequency to 43 and 49 cm^−1^, and *ν*_1_ becomes stronger at 33 cm^−1^ compared with *ν*_2_ and *ν*_3_ (upper panel of Fig. 5b). The peak *ν*_2_ may be the shoulder that remains at 11 K. The spectral change with decreasing the dielectric constant from 15.20 to 8.33 well corresponds the temperature-dependent spectral change from 200 K to 11 K. Unlike the THz spectrum of polymers^42^, DMAPS studied here is a monomer molecule, so the possibility of partial crystallization like in an amorphous one-dimensional long polymer chain is excluded. An observed background growing monotonically from 20 to 60 cm^−1^ is the characteristics of amorphous THz spectra^28^.

### Vibrational modes giving temperature dependence

**Figure 6:**
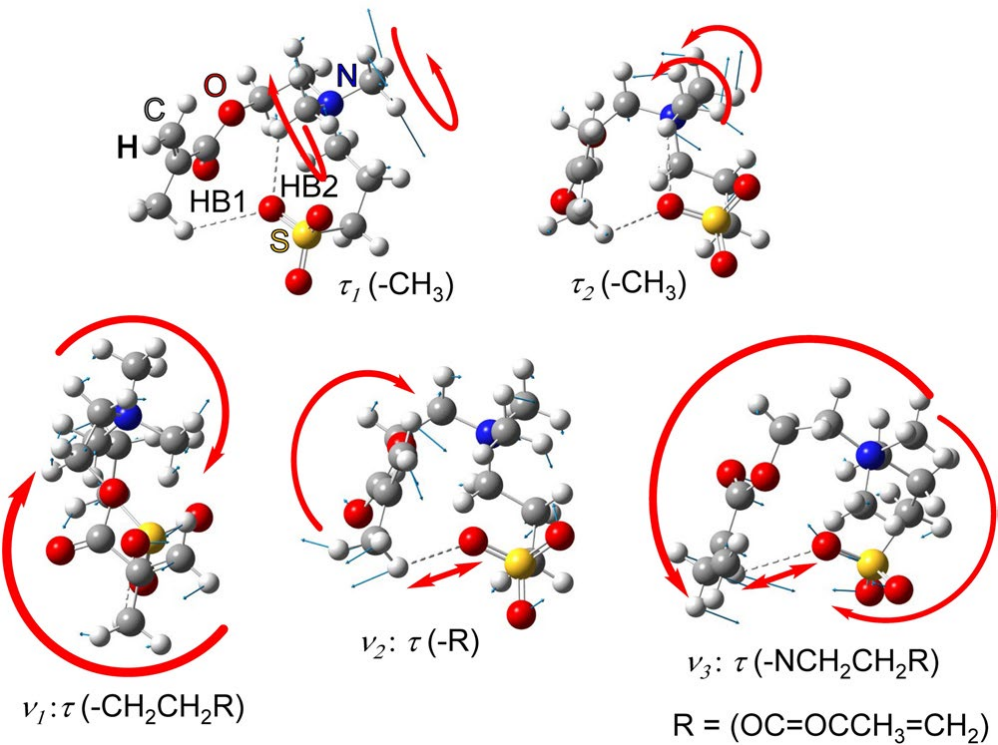
Vibrational mode of two peaks showing a temperature-dependent spectral change in the FIR region of DMAPS (*τ*_*1*_ (-CH_3_) and *τ*_*2*_ (-CH_3_)) and the lowest three vibrations in THz region (*ν*_*1*_, *ν*_*2*_, *ν*_*3*_).

The vibrational modes of DMAPS showing a temperature-dependent spectral changes in the FIR and THz regions are examined. The peaks at 307 and 314 cm^−1^ observed at 4 K in the FIR region are assigned to the torsion modes, *τ*_*1*_ (-CH_3_) (disrotation of one methyl group and N(MeR1R2) group) and *τ*_*2*_ (-CH_3_) (corotation of two methyl groups on a nitrogen atom) (Fig. 6). As shown in Table 1, HB2 is a typical O…H bond between methyl hydrogen and SO_3_ oxygen in both the low-temperature model (*ε* = 8.33) and high-temperature model (*ε* = 15.20). The strength of HB2 gradually decreases as the dielectric constant increases. On the other hand, HB1 makes weak hydrogen bond at the VDW limit in low-temperature model, but not in high-temperature model. In low-temperature mode (*ε* = 8.33), the frequency and intensity of *τ*_*1*_ (-CH_3_) and *τ*_*2*_ (-CH_3_) increase due to the increase of HB2 bond strength. Therefore, totally four peaks including *τ*_*1*_ (-CH_3_) and *τ*_*2*_ (-CH_3_) were observed below 100 K. Mode *τ*_*2*_ (-CH_3_) includes movement in the HB1 bonding direction, but not *τ*_*1*_ (-CH_3_). The cleavage of HB1 at *ε* = 15.20 (high-temperature model) allows the O atom of the SO_3_ group to move without constraint from the HB1 bond, and only the intensity of *τ*_*2*_ (-CH_3_) increases. Thus, totally three peaks were observed above 150 K.

Three peaks observed in the THz spectrum are assigned to three torsion modes (*ν*_1_, *ν*_2_, and *ν*_3_ in Fig. 6). Two of the three modes, *ν*_2_ and *ν*_3_, contain stretching vibrations of the HB1 hydrogen bond. As shown in Table 1, HB1 forms a weak hydrogen bond at *ε* = 8.33 and not at *ε* = 15.20. Thus, with the formation of HB1 hydrogen bonding at *ε* = 8.33, the frequencies of *ν*_2_ and *ν*_3_ increase.

## Discussion

**Figure 7:**
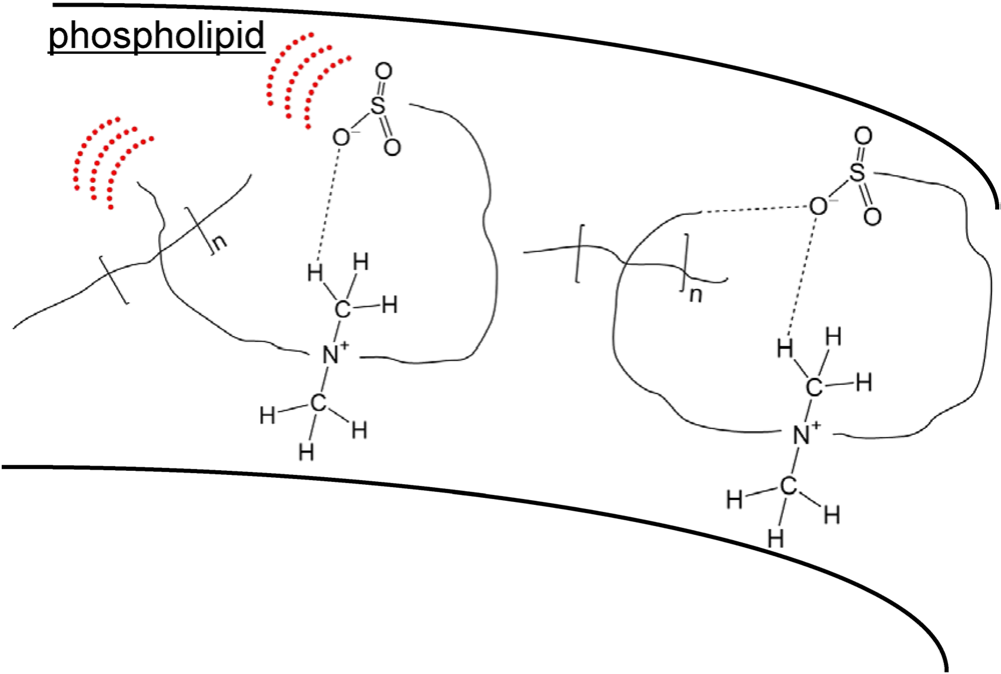
Stimuli-sensitive biocompatible DMAPS polymers that change the form depending on the environment.

Our current work has succeeded in assessing the VDW interaction that converts DMAPS from the thermal-motion form to the hydrogen-bonded form shown in Fig. 1. The conversion between the two forms was obtained clearly as a spectral change in the THz spectral region with a definite isosbestic point indicating a change from one absorption peak to the other. Temperature-dependent spectral change occurred between 150 and 100 K which corresponds to nearly the VDW bonding energy (<240 K). The spectral change by cooling was interpreted as the decrease of dielectric constant due to the reduced mobility at low temperature. Present results suggest that the stimuli-sensitive biocompatible DMAPS polymers change their form depending on the environmental temperature and dielectric constant via the formation of weak hydrogen bond (Fig. 7). The dielectric constants, 8.33. and 15.20 used in the present study are within the range of the dielectric constant of proteins and phospholipids (4~20).

We also succeeded in obtaining distinct THz peaks from completely amorphous DMAPS powder. Most biological materials act in amorphous form, and crystals are rare. Generally, a crystal has a sharp THz peak, and the amorphous form has no feature in the THz spectrum. On the other hand, DMAPS studied here gave THz peaks even though it is not crystalline. It is important to distinguish between the absence of a THz peak and the presence of a very weak and/or broad THz peak. Our previous THz spectroscopic study on crystalline sample^27^ has found that the chain torsion mode is present in the THz energy region. Although there is a crystal field effect, it is expected that vibrational absorption by the torsion mode of the methacryloyl moiety exists near this region. The problem is presumed to be a very low intensity of the vibrational absorption. To overcome this problem, we measured the sample without using a filling material such as polyethylene to dilute the sample. Regarding filling materials, there are also noticeable points in the synchrotron FTIR microspectroscopy measurements in our present study. KBr is normally used as a filling material in FTIR measurements but is not a suitable filling material for DMAPS because KBr may interact with anion and cation pairs in electrically neutral zwitterionic betaine monomer such as DMAPS. Synchrotron FTIR microspectroscopy requires no filling material for measurements and can avoid this interaction.

The complicated FIR spectrum of the present amorphous system was found to be well reproduced by incorporating the effect of the surrounding molecules as the dielectric constant. Deduced from the dielectric constant of the analogous, we have investigated nine solvents with dielectric constants from 1.43 to 46.83 (Table 1). From the number of peaks around 300 cm^−1^ and the frequency in the THz region, we finally selected the value 8.33 for the low-temperature model and 15.20 for the high-temperature model. When incorporating a dielectric constant of 8.33 in the calculation, the calculated spectrum reproduces the experimental spectrum for both frequency and intensity, and the frequency deviation is less than 32 cm^−1^.

## Conclusions

Based on measurements of the temperature-dependent behavior of amorphous DMAPS in the FIR and THz spectral regions and DFT calculations with dispersion correction, we have assessed the VDW interactions that convert DMAPS from the thermal-motion form to the hydrogen-bonded form. In order to treat VDW interactions very accurately, we used the dispersion correction method that provides the accuracy for weak interactions comparable to CCSD(T) that can attain a consistently accurate description of VDW interactions. We found a very weak intramolecular hydrogen bond at the VDW limit formed below 100 K, and the contact distance is calculated to be 2.77 Å. The formation of very weak hydrogen bonds critically causes a change in the number of peaks around 300 cm^−1^ in the FIR spectral region and a high frequency shift for certain modes in the THz spectral region. The present finding of very weak intramolecular hydrogen bonds at the VDW limit in a biocompatible compound leads to the elucidation of the role of VDW interactions in the function of thermal stimuli-sensitivity and biocompatibility.

## Methods

### Materials

DMAPS (3-[dimethyl (2-methacryloyloxyethyl) ammonium] propane sulfonate) was purchased from Sigma-Aldrich (St. Louis, MO) and measured in both synchrotron FTIR microspectroscopy and terahertz time-domain spectroscopy (THz-TDS) without further purification.

### Synchrotron FTIR microspectroscopy

Synchrotron FTIR spectra in the range of 140–700 cm^−1^ were collected with a resolution of 2 cm^−1^ at an infrared beamline BL43IR of the SPring-8 synchrotron radiation (SR) facility (Hyogo, Japan). An FTIR (BRUKER IFS120HR) spectrometer was used with IR-SR as an infrared source. The powder sample dissolved in desalinated water was dropped on a silicon substrate and dried, then inserted into the cryostat and evacuated. The spectrum was the same, even when the powder sample was placed directly on a silicon substrate without dissolving in desalinated water and was inserted into the cryostat and measured. By evacuation it is estimated that the water disappears completely during the measurement. The frequency of each peak in Figs. 2 and 3 was evaluated by fitting the spectrum with a Lorentz function.

### THz-TDS

THz spectra from 20 to 60 cm^−1^ were measured with a Tochigi Nikon RT-20000 THz-TDS with a resolution of 0.8 cm^−1^ at 11–296 K. Powder samples were placed directly on the sample holder with Kapton tape on both sides. We confirmed that the Kapton tape has no absorption in the THz spectral region.

### DFT calculations

To analyse the vibrational absorption spectrum of a fully amorphous powder sample, DFT calculations of gas-phase molecules surrounded by themselves were performed with the Gaussian 09 and 16 software packages^43^. We utilized a hybrid Becke-type three-parameter exchange functional^44^ paired with the gradient-corrected Lee, Yang, and Parr correlation functional (B3LYP)^45,46^ and the aug-cc-pVTZ basis set^47–51^. The Petersson-Frisch dispersion model from the APDF functional was used for dispersion correction^52^. This model provides comparable weak interaction accuracy to that of CCSD(T)/aug-cc-pVTZ, which can attain a consistently accurate description of VDW interactions. The geometric parameters were fully optimized, and the optimized structures were confirmed to have no imaginary frequency. To select stable conformers, the Gibbs free energy at 298 K was calculated with highly accurate CBS-Q (see Supplementary Table S1 for thermodynamic data). The effect of the surrounding molecules was incorporated as a solvent under the PCM model using the integral equation formalism variant (IEF-PCM)^36^. To investigate the effect of various solvents and their dielectric constants, 9 solvent keywords accepted with SCRF = Solvent option were used. The cavity type used for PCM calculations was a scaled VDW which places a sphere around each solute atom with the universal force field (UFF) radii^53^ scaled by a factor of 1.1. The scaling factor depends on solvent, solute^36^, and the definition of atomic radii^54,55^. Examining the effects of the scaling factors, we found that the results with factor 1.1 fit well to the FIR spectrum at 4 K and can account for temperature-dependent spectral changes. To obtain the reliable result for very low frequency as in THz region, a superfine integration grid was used.

We investigated all possible conformations of the methacryloyl moiety of the DMAPS molecule for the propane sulfonate moiety in the ring form at the B3LYP/cc-pVTZ to elucidate the conformation of DMAPS in the amorphous state studied here (see Supplementary Fig. S2). For the propane sulfonate moiety, we confirmed that the ring form connected between positive nitrogen and negative oxygen is much more stable by 18 kcal/mol than the linear form without N^+^–O^−^ connection at the B3LYP/cc-pVTZ + ZPE level with dispersion correction (D3 version of Grimme’s empirical dispersion with the original D3 damping function)^56^. We have obtained 24 conformations, starting from 27 conformations of methacryloyl moiety with a transoid -C(=O)-C(CH_3_=CH_2_) at the terminal.

## Supplementary information

Assessment of the VDW interaction converting DMAPS from the thermal-motion form to the hydrogen-bonded form

## Data Availability

The data generated or analysed during this study are available from the corresponding author on reasonable request.
