# Supplementary material for: Assessment of the VDW interaction converting DMAPS from the thermal-motion form to the hydrogen-bonded form
Source: Sci Rep. 2019 Sep 11;9:13104. doi: 10.1038/s41598-019-49352-1 (PMC6739504; doi:10.1038/s41598-019-49352-1)
Supplement: Supplementary file 1 — Assessment of the VDW interaction converting DMAPS from the thermal-motion form to the hydrogen-bonded form [file 41598_2019_49352_MOESM1_ESM.pdf]

Supplementary Information

Assessment of the VDW interaction converting DMAPS from the thermal-motion form to the hydrogen-bonded form

Masae Takahashi, Hiroshi Matsui, Yuka Ikemoto, Makoto Suzuki & Nobuyuki Morimoto

Table S1. Thermodynamic data of DMAPS conformers (in kcal/mol)[a]

Conformers[b]	B3LYP[c]	CBS-Q
111	0.00	0.00
112	0.62	-3.08
121	1.02	-0.88
123	1.10	1.08
132	-0.05	-0.94
133	-0.51	-1.19
211	1.12	3.16
212	1.33	1.79
213	1.62	5.09
221	4.01	5.75
222	3.14	3.28
223	1.96	4.89
231	1.80	5.50
232	2.59	3.79
233	2.05	4.71
311	1.69	2.08
312	1.08	-2.25
313	1.63	-3.18
321	2.18	5.40
322	3.24	4.46
323	4.05	4.44
331	1.75	4.35
332	1.63	1.92
333	1.46	2.16

[a] Relative Gibbs free energies at 298 K. [b] See Figure S1 for the numbering of conformers. [c] B3LY/cc-pVTZ with dispersion correction.

Table S2. Vibrational frequencies of DMAPS

Calc.[a]		Expl[b]/4K	
Frequency/cm^{-1}	Intensity/km mol^{-1}	Frequency/cm^{-1}	$\Delta_{calc-expl}$/cm^{-1}
166	12.62	157	9
197	7.88	189	8
204	1.18	200	4
227	1.13	223	4
253	4.05	274	-21
286	3.15	307	-21
301	1.72	314	-13
314	7.85	330	-16
331	11.18	343	-12
343	1.88	375	-32
373	2.75	396	-23
419	3.53	429	-10
461	1.17	461	0
494	22.34	507	-13
502	21.53	523	-21
508	20.25	528	-20
561	7.06	587	-26
568	39.48	568	0
595	1.34	601	-6
655	27.41	670	-15

[a] Calculations at the B3LY/aug-cc-pVTZ level under the PCM model using ε = 8.33 and superfine grid of integral for the DMAPS conformer shown in Fig. 4. [b] Present work at 4 K.

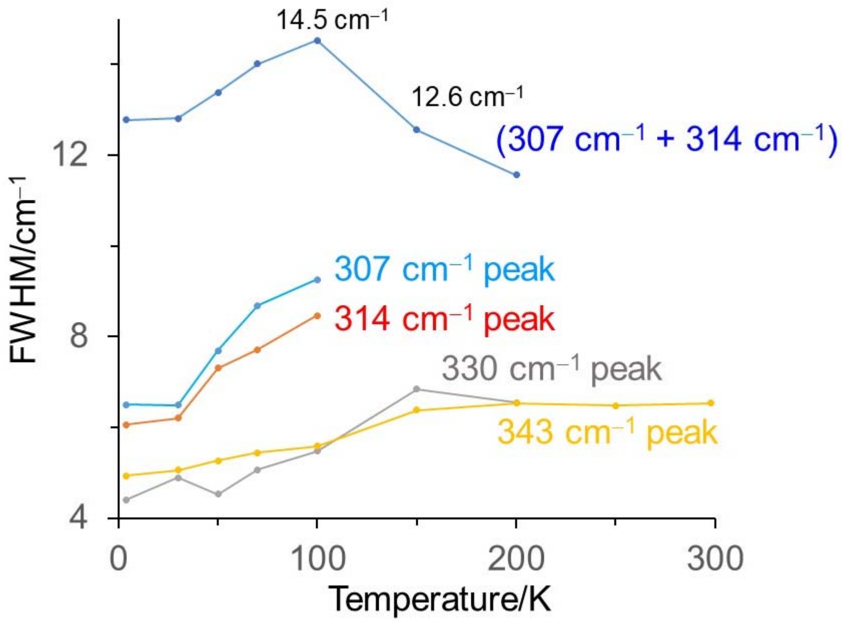

Figure S1. Temperature dependence of FWHM (full width at half maximum) of four peaks observed around 300 cm^{-1} in FIR spectrum. FWHM was evaluated by fitting the spectrum with a Lorentz function for 4, 30, 50, 70, 100, 150, and 200 K, and a Gaussian function for 250 and room temperature. The COD (coefficient of determination) values are more than 0.99 for all fitting data. The FWHM value of 307 cm^{-1} + 314 cm^{-1} was estimated as the following equation.

((FWHM of 307 cm^{-1} peak) + (FWHM of 314 cm^{-1} peak))/2 + (frequency difference between 307 cm^{-1} peak and 314 cm^{-1} peak).

This corresponds to FWHM of two overlapped peaks.

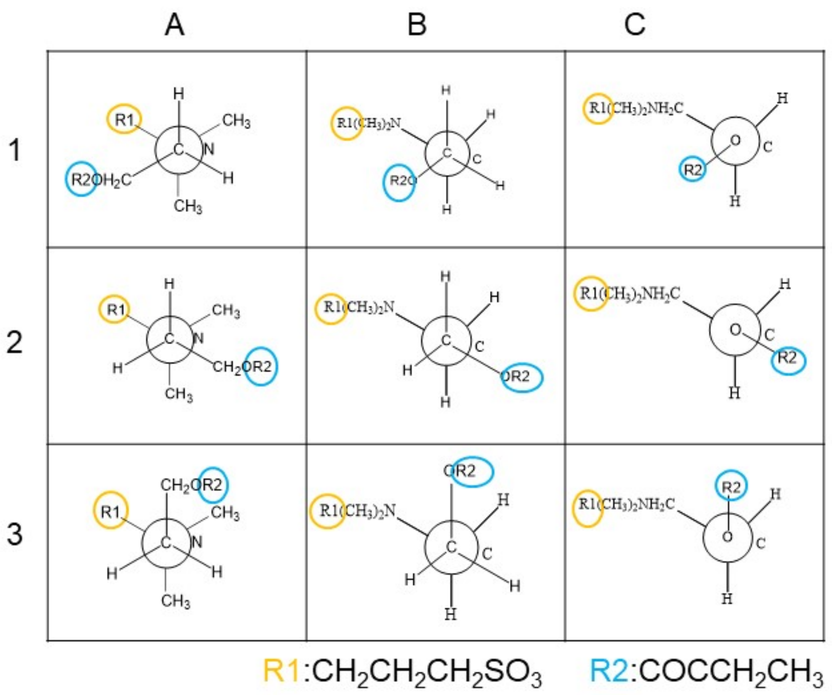

Figure S2. Numbering the 27 conformations, ABC, of DMAPS.
